# Illumina MiSeq and iSeq platforms yield comparable results for viral genomic sequencing

**DOI:** 10.1016/j.jviromet.2025.115202

**Published:** 2025-06-13

**Authors:** Darlene D. Wagner, Grace Nabakooza, Nehalraza Momin, Rachel L. Marine

**Affiliations:** a Eagle Global Scientific LLC, contracting agency to the Centers for Disease Control and Prevention, Atlanta, Georgia; b Division of Viral Diseases, Centers for Disease Control and Prevention, Atlanta, Georgia

**Keywords:** Next-generation sequencing (NGS), Sequencing Quality, Illumina, Cost Comparison, RNA viruses

## Abstract

Illumina MiSeq and iSeq are widely used short-read next-generation sequencing (NGS) platforms. The MiSeq instrument is specialized for mid-range throughput, while the iSeq instrument has lower throughput but is less expensive and simpler to operate. Several studies have compared Illumina platforms for sequencing of a variety of specimen types, but very few have quantified differences in sequencing quality, particularly for viruses. This study compared read quality, single nucleotide polymorphism (SNP) calling, and assembly metrics for SARS-CoV-2, norovirus, and poliovirus samples sequenced on both platforms. MiSeq and iSeq trimmed reads exhibited equivalent percentage of bases ≥ Q30 (% ≥ Q30) and equivalent reference mapping percentages. SNP concordance rates between the two platforms were 41 out of 43 (95.3 %) for SARS-CoV-2, 1628 out of 1633 (99.7 %) for norovirus, and 9 out of 11 (81.8 %) for poliovirus. Within each of the three virus groups, MiSeq and iSeq assemblies had equivalent N50 and maximum contig lengths. Despite platform-specific differences in the sequencing chemistry and flow cell design, MiSeq and iSeq offered comparable data quality, indicating that using either platform should produce concordant results for viral genomic sequencing.

## 1. Introduction

Next-generation sequencing (NGS) technologies have facilitated tracking viral disease outbreaks, vaccine development, surveillance, and general virology research (Barzon et al., 2013; Berry et al., 2019; MacCannell, 2016). For example, NGS platforms such as those developed by Illumina and Oxford Nanopore Technologies enabled epidemiological tracking and diagnosis of SARS-CoV-2 from early stages of the COVID-19 pandemic (Chiara et al., 2020). Illumina systems continue to be the predominant short-read NGS technology, with the MiSeq considered a standard platform for mid-range throughput (Berry et al., 2019). In comparison, the Illumina iSeq platform, with its lower instrumentation cost, is well-suited for laboratories new to NGS or in locations where on-site maintenance is difficult to arrange (Colman et al., 2019; Mitchell et al., 2022; Poates et al., 2022).

In addition to cost, sequencing laboratories should also consider standards for quality assurance and benchmarks for quality metrics. Different NGS platforms exhibit distinct profiles of read quality and error distribution (Jünemann et al., 2013; Loman et al., 2012; Mellmann et al., 2017). MiSeq and iSeq have underlying differences in sequencing chemistry, imaging process, and flow cell technology. MiSeq employs a non-patterned flow cell with four-channel chemistry (four dyes and four imaging steps per cycle), whereas the iSeq platform utilizes a patterned flow cell with single-channel sequencing (i.e., one-dye with two imaging steps per cycle, (https://knowledge.illumina.com/)). Despite these differences, MiSeq and iSeq share key features canonical to Illumina sequencing, including the Illumina proprietary sequencing-by-synthesis procedure (Shendure and Ji, 2008; Shokralla et al., 2012). Given the differences and similarities between MiSeq and iSeq, systematic comparisons of these platforms’ quality metrics are needed. While several studies have been published comparing MiSeq and iSeq for sequencing of various specimen types (Colman et al., 2019; Daca-Roszak et al., 2024; Foster et al., 2022; Goya et al., 2018; Nakao et al., 2021; Ranasinghe et al., 2022; Román-Reyna et al., 2021; Salamon et al., 2022; Trees et al., 2023; Uelze et al., 2020), only one of these studies (Foster et al., 2022) focused on sequencing of a viral pathogen and only Uelze et al. (2020) compared quality metrics between the platforms. In the current study, read quality and assembly metrics for the MiSeq and iSeq platforms were compared for shotgun sequencing of poliovirus and targeted sequencing of SARS-CoV-2 and norovirus.

## 2. Materials and methods

### 2.1. Sample extraction and preparation

Eight SARS-CoV-2 nasopharyngeal (NP) swab samples, seven norovirus stool samples, and eight (originally thirteen) poliovirus isolates were processed for sequencing.

Norovirus RNA was extracted using the MagMax-96 Viral RNA Isolation kit (Applied Biosystems, Waltham, MA, USA) with a KingFisher Flex Purification System according to manufacturer’s instructions (Thermo Scientific, Waltham, MA, USA). Norovirus RNA was treated with dsDNase (Thermo Scientific) for 2 min at 37°C to remove genomic double-stranded DNA. The samples were then treated with 0.1 M DTT for 5 min at 55°C to inactivate the dsDNase.

Poliovirus cell culture isolates spotted onto FTA cards were extracted using a previously detailed process (Montmayeur et al., 2017; Ng et al., 2012). Total nucleic acids were extracted from FTA eluate using the QIAamp Viral RNA Minikit (Qiagen, Germantown, MD, USA) without carrier RNA and eluted with 50 μl AVE buffer solution.

SARS-CoV-2 RNA was extracted from NP swabs in viral transport medium using EZ1 DSP Virus Kit on the EZ1 Advanced XL instrument (Qiagen) following manufacturer’s instructions.

### 2.2. Reverse transcription and random/targeted amplification

Norovirus RNA was first denatured in a reaction containing 6 μL of water, 2 μL of 100 μM reverse primer TX30SXN (Katayama et al., 2002; Kojima et al., 2002; Parra et al., 2017), 5 μL dsDNase-treated RNA, and 1 μL of 10 mM dNTPs for 5 min at 64°C then for 2 min at 4°C. cDNA was generated using the Maxima H Minus First Strand cDNA Synthesis Kit (Thermo Fisher) under the following thermocycler conditions: 1 h at 50°C, 5 min at 85°C, 2 min at 4°C. Full genome amplicons were generated using SequelPrep Long PCR Kit (Applied Biosystems) with primers targeting the conserved regions of the 5’- and 3’- ends of norovirus GII genotypes (Katayama et al., 2002; Kojima et al., 2002; Parra et al., 2017), under the following thermocycler conditions: 94°C for 2 min, 9 cycles [94°C for 10 sec, 55° for 30 sec, 68°C for 9 min], 29 cycles [94°C for 10 sec, 55°C for 30 sec, 68°C for 9 min 20 sec, with a 20 s extension per cycle], 72°C for 5 min, and hold at 11°C. Amplicons were purified using ExoSAP-IT PCR Product Cleanup kit (Applied Biosystems).

Poliovirus RNA was reverse transcribed using the qScript Flex cDNA kit (QuantaBio, Beverly, MA, USA). Briefly, 5 μL viral RNA was added to an aliquot containing QuantaBio qScript cDNA kit 5 × random primer and 0.1 × oligo dT, followed by heat denaturation for 5 min at 65°C and chilled for 2 min at 4°C. The denatured samples were then added to a master mix containing qScript reverse transcriptase and Vent (exo^−^) DNA polymerase (NEB, Ipswich, MA, USA) and incubated under the following conditions: 5 min at 25°C, 30 min at 42°C, 10 min at 95°C, 2 min at 4°C, and 30 min at 72°C. Excess primers and unincorporated deoxynucleotides were then removed by using the ExoSAP-IT PCR Product Cleanup Kit (Applied Biosystems).

For SARS-CoV-2 samples, first strand cDNA synthesis was performed using Superscript IV reverse transcriptase and 25 μM random hexamer primers under the following thermocycler conditions: 10 min at 25°C, 10 min at 50°C, and 10 min at 80°C. SARS-CoV-2 amplicons were generated using a 4-pool multiplex PCR approach adapted from a 6-pool procedure described previously (Paden et al., 2020). Six microliters of cDNA were added to each pool of primal PCR master mix, containing 10 μM of primer pairs for each pool and NEBNext Q5 Hot Start HiFi PCR master mix, and reacted under the following thermocycler conditions: 30 s at 98°C, 40 cycles of 98°C for 15 s and 65°C for 5 min. Multiplex PCR reactions were pooled and purified using 1.8 × AMPure XP (Beckman Coulter, Brea, CA) bead cleanup, and concentrations were determined using the Quant-IT 1 × dsDNA HS kit (Invitrogen).

### 2.3. Library construction and sequencing

DNA libraries were constructed on a Zephyr G3 NGS workstation (PerkinElmer) with 40 ng of cDNA/amplicon pool using the DNA Library Prep Kit (Illumina, San Diego, CA) with halved reactions following manufacturer’s protocol. Libraries were purified with AMPure XP beads at 1.8 × ratio. The purified samples were quantified on the Biotek Synergy LX plate reader (Agilent Technologies, Santa Clara, CA) with a Quant-IT 1 × dsDNA HS kit and visualized with a TapeStation 4200 (Agilent Technologies). For sequencing, samples were split into two library pools: the first pool contained the norovirus and SARS-CoV-2 samples and the second pool contained the poliovirus samples. These pooled libraries were sequenced on both MiSeq and iSeq platforms using 300-cycle (2 ×150 base pairs (bp), paired end) MiSeq v2 Micro Kit and iSeq i1 reagent cartridges, respectively. Sequencing time for both platforms is approximately 19 h. Bases were called on the MiSeq using Real Time Analysis (RTA) version 1.18.54.4 and iSeq using RTA version 2.12.6.0. Five out of the original 13 poliovirus samples sequenced were ultimately excluded from downstream analysis and inclusion in the study due to a prior data sharing agreement with international laboratories.

### 2.4. Bioinformatics analyses

Raw MiSeq and iSeq reads underwent no other preprocessing/modification on board their respective instruments. Raw reads were processed using Trimmomatic to remove contaminating adapters and low quality bases (Bolger, Lohse, and Usadel, 2014), with maximum mismatches set to 4, alignment score (Q score) threshold for clipping paired ends set to 30, alignment score threshold for single ends and adapter set to 7, minimum adapter length set to 8, both reads retained (True), sliding window size set to 4, average window size Q-score set to 15, and minimum read length set to 50 bp.

Read counts before and after trimming were calculated using the Perl script, friends_rachel.pl, available from github.com/lskatz/ROSS. The percentage of reads with a PHRED score ≥ 30 (% ≥ Q30) was calculated using a custom Python script, Q30andDoubleShuffledFastq.py, available from github.com/darlenewagner/NGS_Plot_Widgets. To expedite mapping and SNP calling and to ensure reproducibility of parameters, Bowtie2 v2.3.5.1 (Langmead and Salzberg, 2012), samtools v1.15.0 (Danecek et al., 2021; Li et al., 2009), and bcftools v1.10.0 (Danecek et al., 2021) were called from a custom workflow in Nextflow v21.04.3 ((Di Tommaso et al., 2017); github.com/darlenewagner/nextflowViral). SNP depth and quality were specified within the Nextflow pipeline by setting the following parameters for *bcftools mpileup*: maximum depth at 35,000 ×, minimum mapQ quality at 20, minimum BAQ quality at 20, and at least 25 × coverage for each SNP position.

The SARS-CoV-2 reference sequence used for read mapping was GenBank Accession MN908947 (Wuhan-Hu strain, (Wu et al., 2020)). Norovirus GII.4 Sydney (GenBank Accession OL913976) was used as the reference for norovirus datasets, and engineered live attenuated human poliovirus 2 (GenBank Accession MZ245455) was used as the reference for poliovirus datasets. SNPs sharing identical positions on respective references (i.e., concordant SNPs) were determined by the following formula for each species set

$$C=M_{\text{uniq}}\cap I_{\text{uniq}}=\left(M_{1}\cup M_{2}\cup \ldots \cup M_{n}\right)\cap \left(I_{1}\cup I_{2}\cup \ldots \cup I_{n}\right)$$

Here, to compute concordance, **C**, the intersection is taken between unique MiSeq positions, denoted by ***M***_***uniq***_, and unique iSeq positions, ***I***_***uniq***_. ***M***_***uniq***_ and ***I***_***uniq***_ are computed by taking the union across samples where each ***M***_***i***_ denotes the ***i***th sample on MiSeq while ***I***_***i***_ denotes the same sample on iSeq.

Average insert lengths (i.e., sequence fragments spanning forward and reverse reads) were calculated as previously described (Wagner et al., 2021). De novo assemblies were performed by using SPAdes (version 3.15.0) with parameters as described previously (Wagner et al., 2022) and contigs were filtered and trimmed to within their respective reference genome lengths using a custom workflow (trimViralNF) with contigs > 200 bp retained. Phyletic composition of unscreened poliovirus reads was quantified by Kraken2 (Wood and Salzberg, 2014).

Wilcoxon rank sum tests were implemented in R v3.6.1. Venn diagrams were plotted with vennDiagramPlotColumn.py (NGS_Plot_Widgets).

### 2.5. Data sharing

Untrimmed MiSeq and iSeq fastq data from SARS-CoV-2, norovirus, and poliovirus samples were masked for human reads using sra-human-scrubber (https://github.com/ncbi/sra-human-scrubber.git) and uploaded to NCBI SRA (PRJNA1041361). No statistical differences between raw reads and human-screened reads were observed for SARS-CoV-2 and norovirus (Supplementary Tables S1 – S2). Untrimmed, unscreened reads from poliovirus samples were equivalent to human-screened reads with respect to % ≥ Q30, N50, and maximum contigs length, but were statistically distinct for read counts, insert lengths, and percentage reads mapped (Supplementary Table S3). In this study, we report on the results of the unmasked data following adapter trimming to account for background nucleic acid in the starting material (Supplementary Tables S4 – S6).

## 3. Results

### 3.1. Read quality

MiSeq and iSeq % ≥ Q30 scores for paired reads were statistically distinct for SARS-CoV-2 samples (p = 0.0078; Supplementary Tables S7 and S8). By contrast, MiSeq and iSeq % ≥ Q30 for paired reads were equivalent for norovirus and poliovirus samples (p ≥ 0.016; Supplementary Tables S9 – S12). When considering forward and reverse reads separately, % ≥ Q30 scores for poliovirus reads were higher on the MiSeq for forward reads and higher for iSeq for reverse reads (p = 0.0078, Table 1, Supplemental S11). The % ≥ Q30 scores for SARS-CoV-2 forward and reverse reads were significantly higher on the iSeq compared to MiSeq. No significant platform-specific trends were observed for forward or reverse norovirus reads, not even prior to trimming (Supplementary Table S13).

Distinct trends in read count, read length, and insert length were observed between MiSeq and iSeq datasets. While raw read counts per sample were usually higher for iSeq than MiSeq (Supplementary Tables S4 – S6), lower percentages of iSeq reads (< 86 %) than MiSeq reads (> 86 %) were retained after trimming (Table 1). Average trimmed read lengths were slightly shorter for iSeq (<116 bp) than for MiSeq (<123 bp) and differed significantly for SARS-CoV-2 and poliovirus (Supplementary Tables S4 and S6). Trimmed and untrimmed insert lengths for MiSeq and iSeq were statistically equivalent (Supplementary Tables S4 and S6).

### 3.2. Read mapping, average coverage and SNPs statistics

Despite read quality differences, trimmed MiSeq and iSeq reads mapped to reference sequences at statistically equivalent rates (Supplementary Table S14), averaging 98.4 % and 98.3 % for SARS-CoV-2 samples (Supplementary Table S8), 70 % and 68.4 % for norovirus samples (Supplementary Table S10), and 26.6 % and 26.9 % for poliovirus samples (Supplementary Table S12), respectively. Average coverage depths for MiSeq and iSeq were similar with ≥ 2,115x for SARS-CoV-2, ≥ 2,519x for norovirus, and ≥ 1,025x for poliovirus (Supplementary Tables S8, S10 and S12). The lower proportion and depth of mapped reads for poliovirus samples were due to the presence of bacterial reads (48 % – 69 %, Supplemental Table S12), since libraries were prepared from random cDNA (i.e., shotgun sequencing) rather than targeted viral amplicons.

SNP positions were highly concordant between MiSeq and iSeq mapped reads. A total of 42 unique SNPs were detected across MiSeq and iSeq trimmed SARS-CoV-2 reads; 41 of these SNPs (97.6 %) were concordant between the two platforms (Fig. 1A). For norovirus MiSeq and iSeq reads, there were a total of 1630 unique MiSeq SNPs and 1631 unique norovirus iSeq SNPs, of which 1628 (99.7 %) were concordant (Fig. 1B). Trimmed poliovirus reads yielded 11 unique MiSeq SNPs and 9 unique iSeq SNPs of which 9 SNPs were concordant (81.8 %, Fig. 1C). The two discordant poliovirus SNPs occurred only in the MiSeq reads at positions 1369 and 4502 on MZ245455.1 and had quality scores < 65 (on a scale of 0 – 228).

### 3.3. De novo assembly quality

After read trimming, MiSeq and iSeq assemblies were statistically equivalent across all three viral datasets (Supplementary Tables S8, S10, S12, and S14). The longest contig per sample (i.e., maximum length) for SARS-CoV-2 assemblies ranged from 11 % to 99.7 % the length of MN908947 (29,903 bp); maximum length of norovirus assemblies ranged from 7 % to 100 % the length of OL913976 (7560 bp). The poliovirus maximum lengths were 19–99.7 % the length of the genome of human poliovirus 2 (MZ245455, 7500 bp). Despite the wide range in their breadth of coverage, assembly statistics from MiSeq and iSeq reads were statistically indistinguishable.

### 3.4. Sequencing cost analysis

The estimated cost of sequencing (based on the manufacture’s predicted output for the sequencing kits used in this study) is lower on the MiSeq than iSeq (MiSeq $0.47/million bp; iSeq $0.53/million bp). In this study, the calculated cost per million bp (Mbp) was similar to predictions, with $0.01 lower cost per Mbp for the SARS-CoV-2 and norovirus iSeq runs compared to MiSeq, and $0.05 higher cost per Mbp for the poliovirus iSeq run compared to MiSeq (Table 1).

## 4. Discussion

For paired reads, iSeq and MiSeq % ≥ Q30 scores were equivalent for norovirus and poliovirus samples, whereas those for SARS-CoV-2 samples differed significantly. Previous studies have reported similarly mixed results on quality metric differences between MiSeq and iSeq. In a metagenomic survey of prokaryotes, 300-cycle iSeq % ≥ Q30 exceeded 600-cycle MiSeq % ≥ Q30 by at least 5 percentage points (Salamon et al., 2022). In another bacterial study, the % ≥ Q30 for 300-cycle iSeq reads exceeded 600-cycle MiSeq by only 1 percentage point (Uelze et al., 2020). However, neither of these studies controlled for MiSeq and iSeq read lengths. A more recent bacterial study (replicated across 15 laboratories) comparing at least one 300-cycle MiSeq run versus 300-cycle iSeq found that the % ≥ Q30 of the two platforms were statistically equivalent (Forth et al., 2023). While previous studies have also shown statistically equivalent % ≥ Q30 across MiSeq and iSeq reads (Nakao et al., 2021; Uelze et al., 2020), none have demonstrated such equivalent metrics for viral reads. The current study showed trimmed read length differences between MiSeq and iSeq. Yet, neither read lengths nor insert lengths seemed to affect downstream analyses of the viral sets, same as for bacteria (Wagner et al., 2021).

Downstream analysis results such as SNP identification and *de novo* assembly metrics (N50 and maximum contig lengths) were equivalent between platforms in the current study. This matches a previous study where MiSeq and iSeq reads yielded equivalent SNP results for SARS-CoV-2 and HIV-1 samples (Foster et al., 2022; Mohamed, Boulmé, and Sayada, 2023). A bacterial genomic study comparing MiSeq and iSeq found only one discordant SNP each for *Salmonella enterica* and *Escherichia coli* samples (Forth et al., 2023). In a previous cross-laboratory, cross-platform study, differences in assembly quality were more apparent across distinct laboratories than between different Illumina instruments (Uelze et al., 2020). Another recent cross-laboratory bacterial genome study found similar N50 values across four different Illumina platforms; however, N50 varied with respect to library preparation, especially for Nextera XT given its sensitivity to GC content (Forth et al., 2023). Thus, *de novo* assembly and SNP analyses are likely more greatly influenced by library preparation method than the Illumina platform used for sequencing.

There are three limitations to this study. The viruses used for the comparison study are all positive-sense, single-stranded RNA viruses. However, the only other study to date comparing the iSeq and MiSeq platform for viral sequencing was similarly limited, focusing only on SARS-CoV-2 (Foster et al., 2022). Second, there are likely differences in how % ≥ Q30 is computed across platforms. The quality measure, % ≥ Q30, depends upon PHRED scores. PHRED scores, in turn, vary according to read cycle, GC composition, and read context (DePristo et al., 2011; Ewing and Green, 1998; Ewing et al., 1998; Li, Nordborg, and Li, 2004). PHRED score calculation also depends on the base-calling software of the platform (Cacho et al., 2016; Pereira, Oliveira, and Sousa, 2020). Finally, Illumina has just released a new series of MiSeq instrument, the MiSeq i100, which leverages a patterned flow cell (similar to the iSeq system) and the newest XLEAP-SBS chemistry (www.illumina.com/systems/sequencing-platforms/miseq-i100.html). Moreover, the MiSeq i100 is recommended as the alternative to the iSeq 100 after the latter is phased out in September, 2025 (www.illumina.com/systems/sequencing-platforms/iseq.html). Preliminary estimates on the cost of the instrument indicate that the new instrument is similar in price to the legacy MiSeq system. Given the reproducibility between MiSeq and iSeq platforms, our study suggests that public health laboratories that retain their legacy MiSeq instrument should generate comparable results to MiSeq i100.

Equivalence between MiSeq and iSeq with respect to SNP analysis and assemblies has significant economic implications for laboratories engaged in NGS. In terms of up-front cost, iSeq is a more economic option, being approximately one-fifth the cost of MiSeq as of late 2024. In addition, iSeq offers easier instrument loading with less hands-on time since all reagents needed for sequencing, including reagents for library denaturization, are included in a single cartridge. While the higher cost per Mbp for MiSeq compared to iSeq in the SARS-CoV-2 and norovirus runs was unexpected, a previous study has shown that cost varies widely with number of samples per run. For sequencing of *Mycobacterium tuberculosis* at 30 × genome coverage, MiSeq costs were $60.77 for 11 samples whereas iSeq was only $56.82; yet MiSeq was generally cheaper for 22 or more samples per run (Colman et al., 2019). The MiSeq platform, which provides greater versatility in read length and data output, would be more cost-effective for laboratories that require high sample throughput. MiSeq also enables a broader range of options in number of cycles (read lengths) and data output. Conversely, the ease of operation and reduced instrument cost of the iSeq is ideal for lower-throughput NGS laboratories. Our study indicates viral assemblies are comparable between the two platforms while SNP concordances between platforms is high (except in cases of low SNP quality in the poliovirus set); laboratories should choose the Illumina instrument that best meets their needs based upon budget and needed sequencing output.

## Supplementary Material

Supplementary Materials

## Figures and Tables

**Fig. 1.**
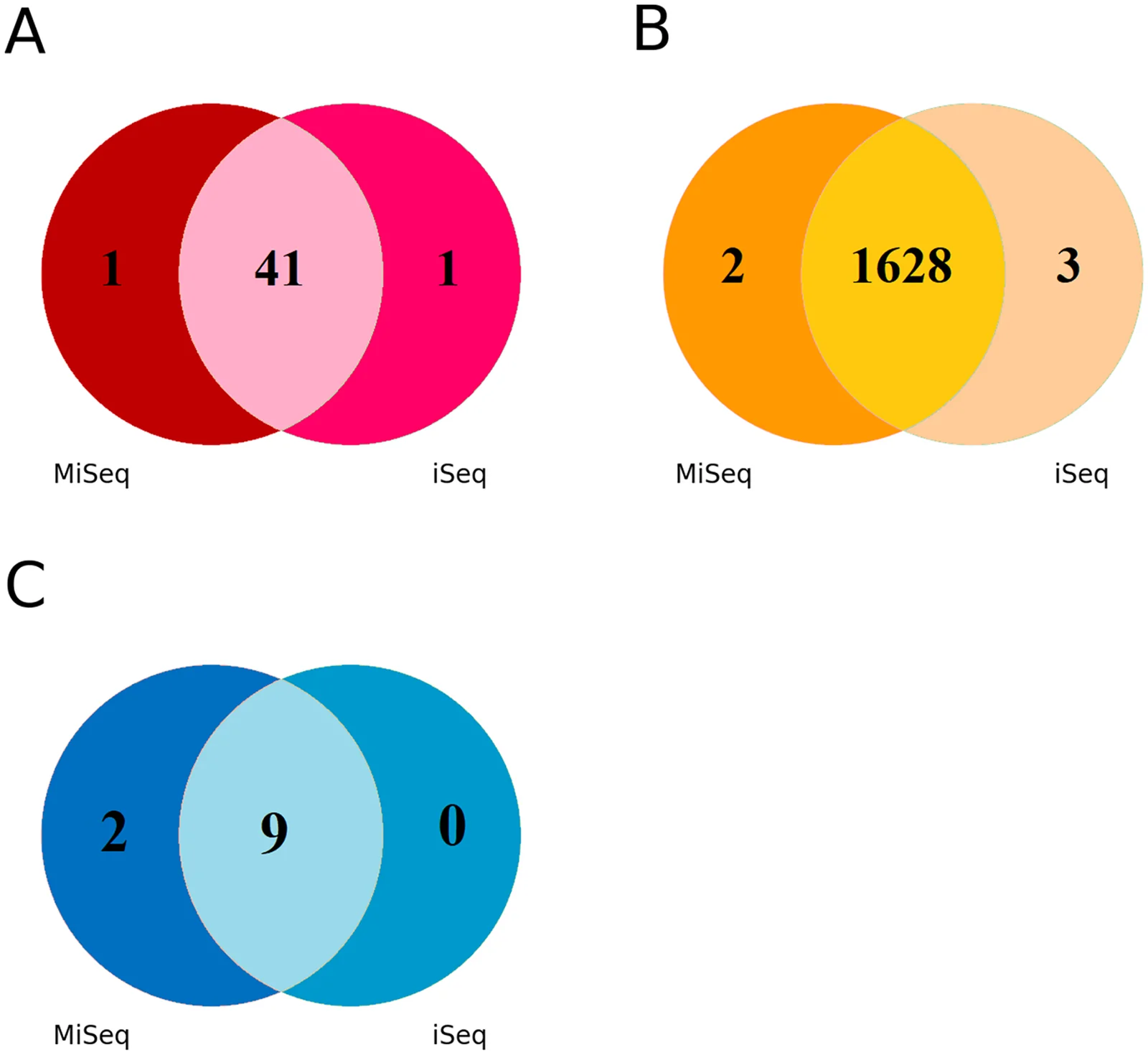
SNP concordance between trimmed MiSeq and iSeq reads. Each comparison shows the discordant MiSeq SNP counts (left), concordant SNP counts (center), and discordant iSeq SNP counts for **A.** SARS-CoV-2, **B.** norovirus, and **C.** poliovirus.

**Table 1:** Read quality, read counts, and cost for SARS-CoV-2, norovirus, and poliovirus samples sequenced on Illumina MiSeq and iSeq platforms. Average % ≥ Q30 are shown for paired reads along with forward and reverse reads separately. Average read counts are shown for raw, unscreened reads (Supplementary Tables S4 – S6) and percentages remaining after trimming are shown in parentheses.

Virus	Sample Count	Platform	Paired % ≥Q30 (average)	% ≥Q30 forward (average)	% ≥Q30 reverse (average)	Average Read Count (% retained reads)	Millions of bp (Mbp)^b^	Cost per Mbp
SARS-CoV–2	8	MiSeq	93.8 – 95.6 (95.0)	95.3 – 96.3 (95.8)	93.0 – 95.1 (94.1)	648,906 (92.0)	1286	$0.44
		iSeq	96.0 – 96.2 (96.1)	96.5 – 96.9 (96.7)	95.2 – 95.9 (95.5)	735,412 (84.3)	1479	$0.43
Norovirus	7	MiSeq	92.0 – 94.3 (93.4)	94.1 – 95.6 (94.9)	88.4 – 94.1 (92.0)	483,460 (86.3)	1286	$0.44
		iSeq	94.9 – 95.4 (95.2)	95.7 – 96.3 (96.0)	93.9 – 94.6 (94.4)	568,145 (79.5)	1479	$0.43
Poliovirus	8^a^	MiSeq	92.7 – 95.7 (94.4)	94.9 – 96.4 (95.9)	89.2 – 95.3 (93.0)	669,556 (92.4)	1305	$0.43
		iSeq	93.3 – 94.1 (93.7)	93.9 – 94.8 (94.3)	92.6 – 93.8 (93.1)	688,853 (85.7)	1343	$0.48

a 13 poliovirus samples used for calculation of millions of base pairs (Mbp) and cost per Mbp because of 5 confidential poliovirus samples excluded from study.

b Combined SARS-CoV-2 and norovirus for calculation of millions of base pairs (Mbp) and cost per Mbp because SARS-CoV-2 and norovirus samples were sequenced on the same run.

## Data Availability

Human screened Illumina reads for all datasets have been submitted to the NCBI SRA database (BioProject PRJNA1041361).

## Appendix A. Supporting information

Supplementary data associated with this article can be found in the online version at doi:10.1016/j.jviromet.2025.115202.
