# Supplementary material for: Illumina MiSeq and iSeq platforms yield comparable results for viral genomic sequencing
Source: J Virol Methods. Author manuscript; Available in PMC 2026 Oct 8. (PMC13647441; doi:10.1016/j.jviromet.2025.115202)
Supplement: Supplementary file 1 [file NIHMS2212288-supplement-Supplementary_Materials.docx]

**Table S1.** MiSeq and iSeq statistics for human-screened, untrimmed reads for SARS-CoV-2 samples. After human screening, MiSeq average insert lengths, % ≥ Q30, reads mapped, N50, and maximum contig lengths were within a statistically equivalent range to raw (not screened, untrimmed) MiSeq reads (Wilcoxon Test, α = 0.01, p = 0.013, p = 0.37, p = 0.74, and p = 0.55). Screened MiSeq read counts did differ statistically from unscreened (p = 0.0078). For human screened iSeq, all six metrics were within a statistically equivalent range to raw reads (Wilcoxon Test, p = 0.014, p = 0.010, p = 0.77, p = 0.64, p = 0.73, and p = 0.55).

| Samples | | Quality | | | | Mapping | | Assembly | |
| --- | --- | --- | --- | --- | --- | --- | --- | --- | --- |
| Platform | Name | Count after screen | % reads remaining | Avg. fragment length | % ≥ Q30 | % Reads Mapped | SNP Count | N50 | Max. Contig |
| MiSeq | SC2_sample_1 | 619,178 | 99.82 | 222 | 89.5 | 99.14 | 38 | 4,044 | 11,332 |
|  | SC2_sample_2 | 633,204 | 99.71 | 223 | 90.9 | 99.09 | 38 | 12,560 | 14,362 |
|  | SC2_sample_3 | 627,366 | 99.21 | 223 | 91.7 | 98.96 | 38 | 19,375 | 19,375 |
|  | SC2_sample_4 | 630,892 | 96.67 | 221 | 89.4 | 98.52 | 36 | 1,110 | 3,820 |
|  | SC2_sample_5 | 698,954 | 95.05 | 219 | 91.2 | 98.01 | 25 | 1,050 | 2,095 |
|  | SC2_sample_6 | 649,270 | 99.76 | 221 | 89.4 | 99.18 | 29 | 1,670 | 4,570 |
|  | SC2_sample_7 | 695,768 | 99.77 | 222 | 89.6 | 99.03 | 35 | 4,418 | 9,573 |
|  | SC2_sample_8 | 564,082 | 99.43 | 221 | 87.8 | 99.15 | 3 | 4,765 | 8,586 |
|  | MiSeq Average | 639,839 | 98.68 | 222 | 89.9 | 98.89 | n/a | 6,124 | 9,214 |
| iSeq | SC2_sample_1 | 678,380 | 99.80 | 218 | 93.3 | 98.34 | 38 | 908 | 1,696 |
|  | SC2_sample_2 | 708,140 | 99.72 | 220 | 93.6 | 98.16 | 38 | 822 | 3,473 |
|  | SC2_sample_3 | 658,384 | 99.18 | 219 | 93.3 | 98.07 | 38 | 877 | 1,870 |
|  | SC2_sample_4 | 820,894 | 96.68 | 218 | 92.6 | 97.32 | 36 | 734 | 2,805 |
|  | SC2_sample_5 | 710,660 | 95.05 | 217 | 93.1 | 96.83 | 25 | 674 | 1,334 |
|  | SC2_sample_6 | 779,772 | 99.74 | 219 | 93.4 | 98.22 | 30 | 780 | 1,361 |
|  | SC2_sample_7 | 696,454 | 99.79 | 219 | 93.4 | 98.27 | 33 | 728 | 2,503 |
|  | SC2_sample_8 | 748,874 | 99.43 | 218 | 92.8 | 98.24 | 3 | 3,133 | 4,280 |
|  | iSeq Average | 725,195 | 98.67 | 219 | 93.2 | 97.93 | n/a | 1,082 | 2,415 |

**Table S2.** MiSeq and iSeq statistics for human-screened, untrimmed reads for norovirus samples. After human screening, MiSeq read counts, average insert lengths, % ≥ Q30, reads mapped, N50, and maximum contig lengths were within a statistically equivalent range to raw reads (Wilcoxon Test, α = 0.01, p = 0.016, p = 0.016, p = 0.022, p = 0.016, p = 0.81, and p = 1.00). Similarly, for human screened iSeq reads, all six metrics were within a statistically equivalent range to raw reads (Wilcoxon Test, p = 0.016, p = 0.034, p = 0.016, p = 0.016, p = 0.58, and p = 0.83).

| Samples | | Quality | | | | Mapping | | Assembly | |
| --- | --- | --- | --- | --- | --- | --- | --- | --- | --- |
| Platform | Name | Count after screen | % reads remaining | Avg. insert length | % ≥ Q30 | % Reads mapped | SNP counts | N50 | Max. contig |
| MiSeq | Noro_sample_1 | 533,374 | 95.97 | 246 | 88.5 | 72.15 | 485 | 583 | 1,772 |
|  | Noro_sample_2 | 593,334 | 93.73 | 229 | 82.8 | 91.90 | 90 | 271 | 523 |
|  | Noro_sample_3 | 353,772 | 81.29 | 232 | 85.5 | 81.07 | 212 | 1,034 | 1,564 |
|  | Noro_sample_4 | 244,010 | 86.72 | 232 | 84.9 | 80.69 | 400 | 863 | 1,564 |
|  | Noro_sample_5 | 397,416 | 98.45 | 233 | 87.7 | 40.65 | 896 | 4,571 | 4,571 |
|  | Noro_sample_6 | 570,482 | 97.75 | 231 | 88.5 | 43.16 | 826 | 321 | 384 |
|  | Noro_sample_7 | 442,026 | 89.92 | 218 | 80.2 | 83.08 | 119 | 265 | 265 |
|  | MiSeq Average | 447,773 | 91.98 | 232 | 85.4 | 70.39 | n/a | 1,130 | 1,520 |
| iSeq | Noro_sample_1 | 538,790 | 96.28 | 225 | 91.0 | 66.62 | 485 | 321 | 321 |
|  | Noro_sample_2 | 625,598 | 93.87 | 220 | 87.8 | 86.35 | 90 | 248 | 341 |
|  | Noro_sample_3 | 495,028 | 84.87 | 220 | 88.0 | 73.11 | 212 | 3,698 | 3,698 |
|  | Noro_sample_4 | 283,416 | 88.92 | 219 | 87.1 | 71.34 | 399 | 763 | 1,434 |
|  | Noro_sample_5 | 525,590 | 98.59 | 219 | 91.1 | 37.33 | 896 | 323 | 323 |
|  | Noro_sample_6 | 610,794 | 97.98 | 219 | 91.2 | 40.39 | 826 | 0 | 0 |
|  | Noro_sample_7 | 638,056 | 92.15 | 222 | 87.1 | 79.20 | 119 | 3,833 | 3,833 |
|  | iSeq Average | 531,039 | 93.24 | 221 | 89.0 | 64.91 | n/a | 1,312 | 1,421 |

**Table S3.** MiSeq and iSeq statistics for human-screened, untrimmed reads for poliovirus samples. After human screening, the range of MiSeq read counts, average insert lengths, and percentage of reads mapped to MZ245455 were statistically distinct from that of unscreened reads (Wilcoxon Test, α = 0.01, p = 0.00024, p = 0.0014, p = 0.00024, p = 0.0089 and p = 0.0061). The range of human-screened iSeq read counts and reads mapped were statistically distinct from unscreened reads (Wilcoxon Test, p = 0.00024 and p = 0.00024) while fragment lengths, % ≥ Q30 and N50 were statistically equivalent (p = 0.027, p = 0.022 and p = 0.033).

| Samples | | Quality | | | | Mapping | | Assembly | |
| --- | --- | --- | --- | --- | --- | --- | --- | --- | --- |
| Platform | Name | Count after screen | % reads remaining | Avg. insert length | % ≥ Q30 | % reads mapped | Count | N50 | Max. contig |
| MiSeq | Polio_sample_1 | 635,058 | 74.59 | 208 | 84.1 | 5.6 | 3 | 7,458 | 7,458 |
|  | Polio_sample_2 | 557,262 | 89.07 | 207 | 86.6 | 5.9 | 4 | 7,442 | 7,442 |
|  | Polio_sample_3 | 465,664 | 93.58 | 207 | 88.8 | 10.0 | 1 | 7,483 | 7,483 |
|  | Polio_sample_4 | 744,330 | 96.64 | 208 | 89.8 | 10.4 | 1 | 7,487 | 7,487 |
|  | Polio_sample_5 | 663,056 | 97.80 | 210 | 87.6 | 14.9 | 1 | 7,442 | 7,442 |
|  | Polio_sample_6 | 667,052 | 89.72 | 209 | 86.8 | 17.6 | 3 | 1,329 | 1,708 |
|  | Polio_sample_7 | 634,020 | 96.10 | 209 | 89.4 | 16.9 | 0 | 7,442 | 7,442 |
|  | Polio_sample_8 | 504,940 | 95.22 | 208 | 85.2 | 10.9 | 1 | 7,442 | 7,442 |
|  | MiSeq Average | 608,923 | 91.59 | 208 | 88.0 | 11.5 | n/a | 6691 | 6738 |
| iSeq | Polio_sample_1 | 683,058 | 75.38 | 212 | 88.3 | 4.5 | 3 | 1,359 | 2,740 |
|  | Polio_sample_2 | 564,072 | 89.44 | 212 | 89.4 | 4.5 | 4 | 289 | 527 |
|  | Polio_sample_3 | 704,362 | 93.72 | 212 | 90.6 | 7.7 | 1 | 369 | 369 |
|  | Polio_sample_4 | 707,910 | 96.69 | 212 | 90.6 | 8.3 | 1 | 358 | 358 |
|  | Polio_sample_5 | 694,926 | 97.87 | 213 | 90.2 | 11.6 | 1 | 246 | 246 |
|  | Polio_sample_6 | 608,568 | 89.97 | 212 | 90.0 | 13.8 | 3 | 292 | 292 |
|  | Polio_sample_7 | 594,718 | 96.30 | 213 | 89.4 | 13.4 | 0 | 242 | 246 |
|  | Polio_sample_8 | 464,238 | 95.50 | 213 | 88.5 | 8.3 | 1 | 340 | 390 |
|  | iSeq Average | 627732 | 91.86 | 212 | 89.7 | 9.0 | n/a | 437 | 646 |

**Table S4.** Read counts and average insert lengths of MiSeq and iSeq unscreened SARS-CoV-2 samples plus read counts, % reads retained, average read length, and average insert length after trimming. Trimmed MiSeq reads (using Trimmomatic) yielded statistically distinct read counts with MiSeq untrimmed reads (Wilcoxon test, α = 0.01 > p = 0.0078). Trimmed iSeq reads also yielded statistically distinct read counts with raw reads (p = 0.0078).

| Samples | | Raw Reads | | Trimmed Reads | | | |
| --- | --- | --- | --- | --- | --- | --- | --- |
| Platform | Name | Read count | Avg. insert length | Read count | % reads retained | Avg. read length | Avg. insert length |
| MiSeq | SC2_sample_1 | 620,266 | 212 | 570,640 | 92.00 | 122 | 140 |
|  | SC2_sample_2 | 635,060 | 212 | 583,358 | 91.86 | 122 | 140 |
|  | SC2_sample_3 | 632,344 | 212 | 582,210 | 92.07 | 123 | 142 |
|  | SC2_sample_4 | 652,654 | 210 | 603,686 | 92.50 | 122 | 139 |
|  | SC2_sample_5 | 735,360 | 209 | 669,224 | 91.01 | 120 | 134 |
|  | SC2_sample_6 | 650,844 | 211 | 601,512 | 92.42 | 122 | 139 |
|  | SC2_sample_7 | 697,376 | 211 | 637,858 | 91.47 | 121 | 138 |
|  | SC2_sample_8 | 567,342 | 209 | 525,088 | 92.55 | 121 | 136 |
|  | MiSeq Average | 648,906 | 211 | 596,697 | 91.99 | 122 | 139 |
| iSeq | SC2_sample_1 | 679,716 | 213 | 573,084 | 84.31 | 114 | 120 |
|  | SC2_sample_2 | 710,144 | 214 | 597,138 | 84.09 | 114 | 120 |
|  | SC2_sample_3 | 663,850 | 213 | 560,884 | 84.49 | 115 | 123 |
|  | SC2_sample_4 | 849,044 | 212 | 717,520 | 84.51 | 114 | 120 |
|  | SC2_sample_5 | 747,678 | 211 | 622,872 | 83.31 | 113 | 118 |
|  | SC2_sample_6 | 781,776 | 213 | 658,798 | 84.27 | 114 | 119 |
|  | SC2_sample_7 | 697,952 | 212 | 590,358 | 84.58 | 115 | 121 |
|  | SC2_sample_8 | 753,136 | 212 | 637,766 | 84.68 | 114 | 118 |
|  | iSeq Average | 735,412 | 213 | 619,803 | 84.28 | 114 | 120 |

**Table S5.** Read counts and average insert lengths of MiSeq and iSeq unscreened norovirus samples plus read counts, % reads retained, average read length, and average insert length after trimming. MiSeq untrimmed read counts were within a statistically equivalent range to trimmed reads (Wilcoxon Test, p = 0.016). Statistical equivalence was also observed between raw and trimmed iSeq read counts (Wilcoxon Test, p = 0.016).

| Samples | | Raw Reads | | Trimmed Reads | | | |
| --- | --- | --- | --- | --- | --- | --- | --- |
| Platform | Name | Read count | Avg. insert length | Read count | % reads retained | Avg. read length | Avg. insert length |
| MiSeq | Noro_sample_1 | 555,784 | 216 | 495,622 | 89.18 | 123 | 155 |
|  | Noro_sample_2 | 632,992 | 218 | 536,458 | 84.75 | 119 | 152 |
|  | Noro_sample_3 | 435,180 | 211 | 384,226 | 88.29 | 122 | 158 |
|  | Noro_sample_4 | 281,364 | 215 | 243,866 | 86.67 | 122 | 157 |
|  | Noro_sample_5 | 403,692 | 215 | 361,282 | 89.49 | 122 | 151 |
|  | Noro_sample_6 | 583,610 | 216 | 522,530 | 89.53 | 122 | 153 |
|  | Noro_sample_7 | 491,600 | 214 | 374,916 | 76.26 | 103 | 102 |
|  | MiSeq Average | 483,460 | 215 | 416,986 | 86.31 | 119 | 147 |
| iSeq | Noro_sample_1 | 559,620 | 213 | 463,686 | 82.86 | 116 | 128 |
|  | Noro_sample_2 | 666,480 | 213 | 533,238 | 80.01 | 111 | 125 |
|  | Noro_sample_3 | 583,306 | 212 | 469,932 | 80.56 | 114 | 130 |
|  | Noro_sample_4 | 318,746 | 213 | 254,930 | 79.98 | 114 | 130 |
|  | Noro_sample_5 | 533,104 | 214 | 434,918 | 81.58 | 114 | 123 |
|  | Noro_sample_6 | 623,356 | 213 | 517,194 | 82.97 | 115 | 126 |
|  | Noro_sample_7 | 692,404 | 224 | 487,458 | 70.40 | 97 | 92 |
|  | iSeq Average | 568,145 | 215 | 451,622 | 79.77 | 112 | 122 |

**Table S6.** Read counts and average insert lengths of MiSeq and iSeq unscreened poliovirus samples plus read counts, % reads retained, average read length, and average insert length after trimming. The range of MiSeq and iSeq untrimmed (raw) read counts were statistically distinct from that of trimmed reads (Wilcoxon Test, p = 0.00024 for both MiSeq and iSeq).

| Samples | | Raw Reads | | Trimmed Reads | | | |
| --- | --- | --- | --- | --- | --- | --- | --- |
| Platform | Name | Read count | Avg. fragment length | Read Count | % reads retained | Avg. read length | Avg. insert length |
| MiSeq | Polio_sample_1 | 851,438 | 204 | 738,144 | 86.69 | 110 | 110 |
|  | Polio_sample_2 | 625,658 | 204 | 553,122 | 88.41 | 111 | 109 |
|  | Polio_sample_3 | 497,594 | 203 | 448,858 | 90.21 | 112 | 114 |
|  | Polio_sample_4 | 770,198 | 205 | 682,328 | 88.59 | 112 | 113 |
|  | Polio_sample_5 | 677,990 | 206 | 605,780 | 89.35 | 113 | 117 |
|  | Polio_sample_6 | 743,512 | 205 | 654,924 | 88.09 | 112 | 115 |
|  | Polio_sample_7 | 659,764 | 205 | 582,804 | 88.34 | 112 | 113 |
|  | Polio_sample_8 | 530,294 | 204 | 465,762 | 87.83 | 111 | 113 |
|  | MiSeq Average | 669,556 | 205 | 619,269 | 88.44 | 112 | 113 |
| iSeq | Polio_sample_1 | 906,142 | 210 | 717,574 | 79.19 | 105 | 102 |
|  | Polio_sample_2 | 630,704 | 210 | 500,228 | 79.31 | 105 | 100 |
|  | Polio_sample_3 | 751,584 | 210 | 603,700 | 80.32 | 106 | 103 |
|  | Polio_sample_4 | 732,176 | 209 | 588,976 | 80.44 | 106 | 104 |
|  | Polio_sample_5 | 710,074 | 210 | 569,418 | 80.19 | 107 | 106 |
|  | Polio_sample_6 | 676,448 | 209 | 539,332 | 79.73 | 106 | 105 |
|  | Polio_sample_7 | 617,564 | 210 | 488,942 | 79.17 | 106 | 104 |
|  | Polio_sample_8 | 486,134 | 211 | 385,630 | 79.33 | 105 | 102 |
|  | iSeq Average | 688,853 | 210 | 590,075 | 79.71 | 106 | 103 |

**Table S7.** Quality, mapping, and assembly statistics for unscreened, untrimmed MiSeq and iSeq SARS-CoV-2 data, with averages shown for each sample. Pairwise % ≥ Q30 (third column) were distinct between MiSeq and iSeq (p = 0.0078 < α = 0.01) while R1 and R2 % ≥ Q30 were equivalent (p = 0.022 and 0.014). SNP counts were based on mapping to the Wuhan-Hu reference for SARS-CoV-2 (MN908947).

| Sample | | Quality | | | Mapping | | Assembly | |
| --- | --- | --- | --- | --- | --- | --- | --- | --- |
| Platform | Name | % ≥ Q30 | Forward % ≥ Q30 | Reverse  % ≥ Q30 | % reads mapped | SNP counts | N50 | Max. contig (bp) |
| MiSeq | SC2_sample_1 | 89.5 | 93.1 | 85.9 | 99.0 | 38 | 26,542 | 26,542 |
|  | SC2_sample_2 | 90.9 | 93.0 | 88.8 | 98.8 | 38 | 9,393 | 10,836 |
|  | SC2_sample_3 | 91.7 | 92.9 | 90.6 | 98.2 | 38 | 2,672 | 5,341 |
|  | SC2_sample_4 | 89.4 | 91.9 | 86.8 | 95.2 | 36 | 1,615 | 3,539 |
|  | SC2_sample_5 | 90.9 | 92.5 | 89.4 | 93.2 | 25 | 846 | 1,752 |
|  | SC2_sample_6 | 89.4 | 92.5 | 86.4 | 98.9 | 29 | 1,538 | 4,442 |
|  | SC2_sample_7 | 89.6 | 93.1 | 86.0 | 98.8 | 35 | 4,419 | 9,569 |
|  | SC2_sample_8 | 87.7 | 91.6 | 83.9 | 98.6 | 3 | 1,918 | 3,998 |
|  | MiSeq Average | 89.9 | 92.6 | 87.2 | 97.6 | n/a | 6,118 | 8,252 |
| iSeq | SC2_sample_1 | 93.3 | 93.9 | 92.6 | 98.2 | 38 | 2,161 | 4,879 |
|  | SC2_sample_2 | 93.6 | 94.4 | 92.8 | 97.9 | 38 | 1,597 | 5,073 |
|  | SC2_sample_3 | 93.3 | 94.1 | 92.5 | 97.3 | 38 | 360 | 1,036 |
|  | SC2_sample_4 | 92.5 | 91.9 | 93.2 | 94.1 | 36 | 454 | 772 |
|  | SC2_sample_5 | 93.0 | 94.1 | 91.9 | 92.0 | 25 | 466 | 754 |
|  | SC2_sample_6 | 93.4 | 93.8 | 93.1 | 98.0 | 30 | 338 | 571 |
|  | SC2_sample_7 | 93.5 | 94.3 | 92.6 | 98.1 | 33 | 446 | 739 |
|  | SC2_sample_8 | 92.8 | 92.1 | 93.5 | 97.7 | 3 | 364 | 565 |
|  | iSeq Average | 93.2 | 93.6 | 92.8 | 96.7 | n/a | 773 | 1,799 |

**Table S8.** Quality, mapping, and assembly statistics for Trimmomatic-trimmed MiSeq and iSeq SARS-CoV-2 data, with averages shown for each sample. SNP counts, SNP quality and depth of coverage estimates were based on mapping to Wuhan-Hu reference, MN908947, 29,903 bp in length. Maximum contig lengths ≥99% the length of MN908947 are underlined in italics.

| Samples | | Quality | | | Mapping | | | Assembly | |
| --- | --- | --- | --- | --- | --- | --- | --- | --- | --- |
| Platform | Name | % ≥ Q30 | Forward % ≥ Q30 | Reverse % ≥ Q30 | % reads mapped | Avg. depth of coverage | SNP counts | N50 | Max. contig (bp) |
| MiSeq | SC2_sample_1 | 94.7 | 96.3 | 93.0 | 99.7 | 2,321x | 38 | 26,432 | 26,432 |
|  | SC2_sample_2 | 95.4 | 96.1 | 94.8 | 99.6 | 2,370x | 38 | 29,809 | *29,809* |
|  | SC2_sample_3 | 95.6 | 96.1 | 95.1 | 99.0 | 2,352x | 38 | 29,814 | *29,814* |
|  | SC2_sample_4 | 95.0 | 95.3 | 94.7 | 96.0 | 2,364x | 37 | 16,188 | 16,188 |
|  | SC2_sample_5 | 95.5 | 96.1 | 94.9 | 94.2 | 2,572x | 26 | 1,084 | 3,313 |
|  | SC2_sample_6 | 95.1 | 95.6 | 94.7 | 99.7 | 2,447x | 29 | 4,626 | 7,269 |
|  | SC2_sample_7 | 94.7 | 96.3 | 93.0 | 99.7 | 2,595x | 35 | 29,790 | *29,790* |
|  | SC2_sample_8 | 93.8 | 94.8 | 92.9 | 99.3 | 2,127x | 3 | 15,354 | 15,354 |
|  | MiSeq Average | 95.0 | 95.8 | 94.1 | 98.4 | 2,394x | n/a | 19,137 | 19,746 |
| iSeq | SC2_sample_1 | 96.1 | 96.8 | 95.3 | 99.7 | 2,178x | 38 | 29,811 | *29,811* |
|  | SC2_sample_2 | 96.2 | 96.9 | 95.5 | 99.6 | 2,267x | 38 | 29,810 | *29,810* |
|  | SC2_sample_3 | 96.0 | 96.7 | 95.3 | 98.9 | 2,115x | 38 | 29,797 | *29,797* |
|  | SC2_sample_4 | 96.1 | 96.5 | 95.6 | 95.8 | 2,621x | 36 | 7,290 | 13,538 |
|  | SC2_sample_5 | 96.0 | 96.7 | 95.2 | 93.9 | 2,230x | 25 | 1,325 | 3,320 |
|  | SC2_sample_6 | 96.1 | 96.7 | 95.6 | 99.6 | 2,502x | 30 | 3,083 | 8,091 |
|  | SC2_sample_7 | 96.1 | 96.8 | 95.4 | 99.7 | 2,244x | 34 | 18,472 | 18,472 |
|  | SC2_sample_8 | 96.1 | 96.4 | 95.9 | 99.2 | 2,412x | 3 | 29,809 | *29,809* |
|  | iSeq Average | 96.1 | 96.7 | 95.5 | 98.3 | 2,321x | n/a | 18,675 | 20,331 |

**Table S9.** Quality, mapping, and assembly statistics for unscreened, untrimmed MiSeq norovirus data, with averages shown for each sample. Pairwise % ≥ Q30 (third column) were equivalent between MiSeq and iSeq (p = 0.016 > α) as were R1 and R2 % ≥ Q30 (p = 0.034 and 0.016). Assembly contigs were screened and trimmed by alignment to the reference (OL913976).

| Samples | | Quality | | | Mapping | | Assembly | |
| --- | --- | --- | --- | --- | --- | --- | --- | --- |
| Platform | Name | % ≥ Q30 | Forward % ≥ Q30 | Reverse % ≥ Q30 | % reads mapped | SNP counts | N50 | Max. contig (bp) |
| MiSeq | Noro_sample_1 | 87.4 | 89.3 | 85.5 | 69.3 | 485 | 358 | 415 |
|  | Noro_sample_2 | 80.2 | 86.4 | 74.1 | 86.2 | 90 | 280 | 523 |
|  | Noro_sample_3 | 82.7 | 84.3 | 81.0 | 65.9 | 213 | 1,649 | 3,697 |
|  | Noro_sample_4 | 81.9 | 85.3 | 78.4 | 70.0 | 400 | 734 | 1,564 |
|  | Noro_sample_5 | 87.1 | 89.0 | 85.2 | 40.0 | 896 | 938 | 1,564 |
|  | Noro_sample_6 | 87.9 | 90.0 | 85.7 | 42.2 | 826 | 284 | 321 |
|  | Noro_sample_7 | 77.7 | 80.6 | 74.9 | 74.9 | 119 | 3,833 | 3,833 |
|  | MiSeq Average | 83.5 | 86.4 | 80.7 | 64.1 | n/a | 1,154 | 1,702 |
| iSeq | Noro_sample_1 | 90.1 | 91.1 | 89.1 | 64.3 | 485 | 241 | 299 |
|  | Noro_sample_2 | 85.5 | 86.2 | 84.8 | 81.2 | 90 | 254 | 274 |
|  | Noro_sample_3 | 86.1 | 86.9 | 85.2 | 62.1 | 212 | 1,405 | 3,698 |
|  | Noro_sample_4 | 84.9 | 86.0 | 83.8 | 63.5 | 399 | 501 | 1,437 |
|  | Noro_sample_5 | 90.7 | 91.2 | 90.1 | 36.9 | 896 | 242 | 242 |
|  | Noro_sample_6 | 90.7 | 91.8 | 89.6 | 39.6 | 826 | 242 | 259 |
|  | Noro_sample_7 | 85.2 | 85.6 | 84.8 | 73.7 | 119 | 3,949 | 3,949 |
|  | iSeq Average | 87.6 | 88.4 | 86.8 | 60.2 | n/a | 976 | 1,451 |

**Table S10.** Quality, mapping, and assembly statistics for Trimmomatic-trimmed MiSeq and iSeq norovirus data, with averages shown for each sample. Depth of coverage, as well as screening and trimming of assembled contigs, was performed based on alignment to norovirus GII reference, OL913976, 7,560 bp in length. Maximum contig lengths ≥99% the length of OL913976 are underlined in italics.

| Samples | | Quality | | | Mapping | | | Assembly | |
| --- | --- | --- | --- | --- | --- | --- | --- | --- | --- |
| Platform | Name | % ≥ Q30 | Forward % ≥ Q30 | Reverse % ≥ Q30 | % reads mapped | Avg. depth of coverage | SNP counts | N50 | Max. contig (bp) |
| MiSeq | Noro_sample_1 | 94.1 | 95.4 | 92.9 | 75.8 | 5,914x | 485 | 3,433 | 3,433 |
|  | Noro_sample_2 | 92.0 | 95.6 | 88.4 | 92.6 | 7,819x | 90 | 6,006 | 6,006 |
|  | Noro_sample_3 | 93.5 | 94.1 | 93.0 | 71.8 | 4,342x | 212 | 1,413 | 2,411 |
|  | Noro_sample_4 | 93.2 | 95.0 | 91.5 | 77.3 | 2,967x | 400 | 610 | 1,558 |
|  | Noro_sample_5 | 94.3 | 94.5 | 94.1 | 44.3 | 2,519x | 896 | 7,559 | *7,559* |
|  | Noro_sample_6 | 94.2 | 95.3 | 93.1 | 45.8 | 3,767x | 826 | 4,243 | 4,243 |
|  | Noro_sample_7 | 92.7 | 94.4 | 91.0 | 82.7 | 4,881x | 120 | 4,346 | 4,346 |
|  | MiSeq Average | 93.4 | 94.9 | 92.0 | 70.0 | 4,601x | n/a | 3,944 | 4,222 |
| iSeq | Noro_sample_1 | 95.4 | 96.3 | 94.6 | 73.9 | 5,077x | 485 | 5,211 | 5,211 |
|  | Noro_sample_2 | 95.4 | 96.3 | 94.5 | 89.3 | 7,055x | 90 | 414 | 552 |
|  | Noro_sample_3 | 95.0 | 95.7 | 94.3 | 71.1 | 4,950x | 213 | 1,408 | 1,706 |
|  | Noro_sample_4 | 95.0 | 96.1 | 93.9 | 73.9 | 2,791x | 399 | 743 | 1,564 |
|  | Noro_sample_5 | 95.2 | 95.7 | 94.6 | 43.6 | 2,809x | 896 | 1,564 | 1,564 |
|  | Noro_sample_6 | 95.4 | 96.2 | 94.5 | 45.1 | 3,456x | 826 | 4,742 | 4,742 |
|  | Noro_sample_7 | 94.9 | 95.4 | 94.3 | 82.2 | 5,936x | 122 | 1,268 | 1,268 |
|  | iSeq Average | 95.2 | 96.0 | 94.4 | 68.4 | 4,582x | n/a | 2,193 | 2,372 |

**Table S11.** Quality, mapping, and assembly statistics for unscreened, untrimmed MiSeq and iSeq poliovirus data, with averages shown for each sample. SNPs were called against MZ245455. Screening and trimming of assembled contigs, was performed based on alignment to the human poliovirus 2 reference, MZ245455, 7,500 bp in length. Maximum contig lengths ≥99% the length of MZ245455 are underlined in italics.

| Samples | | Quality | | | Mapping | | Assembly | |
| --- | --- | --- | --- | --- | --- | --- | --- | --- |
| Platform | Name | % ≥ Q30 | Forward % ≥ Q30 | Reverse  % ≥ Q30 | % reads mapped | SNP counts | N50 | Max. contig (bp) |
| MiSeq | Polio_sample_1 | 90.7 | 91.3 | 77.9 | 4.2 | 3 | 7,458 | *7,458* |
|  | Polio_sample_2 | 85.2 | 91.3 | 82.1 | 5.2 | 3 | 7,442 | *7,442* |
|  | Polio_sample_3 | 90.1 | 90.6 | 87.1 | 9.4 | 1 | 7,483 | *7,483* |
|  | Polio_sample_4 | 85.5 | 91.8 | 87.8 | 10.0 | 1 | 7,487 | *7,487* |
|  | Polio_sample_5 | 86.1 | 91.2 | 84.0 | 14.6 | 1 | 7,442 | *7,442* |
|  | Polio_sample_6 | 84.9 | 91.5 | 82.4 | 15.8 | 3 | 1,329 | 1,708 |
|  | Polio_sample_7 | 90.7 | 91.6 | 87.3 | 16.2 | 0 | 7,442 | *7,442* |
|  | Polio_sample_8 | 90.7 | 90.9 | 79.6 | 10.4 | 1 | 7,442 | *7,442* |
|  | MiSeq Average | 88.0 | 91.3 | 83.5 | 10.7 | n/a | 6,690 | 6,740 |
| iSeq | Polio_sample_1 | 88.6 | 87.7 | 89.4 | 3.4 | 3 | 1,359 | 2,740 |
|  | Polio_sample_2 | 89.5 | 90.1 | 88.9 | 4.1 | 3 | 289 | 527 |
|  | Polio_sample_3 | 90.6 | 90.6 | 90.6 | 7.2 | 1 | 369 | 369 |
|  | Polio_sample_4 | 90.7 | 91.2 | 90.1 | 8.1 | 1 | 358 | 358 |
|  | Polio_sample_5 | 90.2 | 90.8 | 89.5 | 11.4 | 1 | 246 | 246 |
|  | Polio_sample_6 | 90.0 | 90.7 | 89.4 | 12.4 | 2 | 292 | 292 |
|  | Polio_sample_7 | 89.4 | 90.4 | 88.4 | 12.9 | 0 | 242 | 246 |
|  | Polio_sample_8 | 88.5 | 88.0 | 89.0 | 7.9 | 1 | 340 | 390 |
|  | iSeq Average | 89.7 | 89.9 | 89.4 | 8.4 | n/a | 437 | 646 |

**Table S12.** Quality, mapping, and assembly statistics for Trimmomatic-trimmed (unscreened) MiSeq and iSeq poliovirus data, with averages shown for each sample. SNPs were called against MZ245455. Depth of coverage, as well as screening and trimming of assembled contigs, was performed based on alignment to the human poliovirus 2 reference, MZ245455, 7,500 bp in length. Maximum contig lengths ≥99% the length of MZ245455 are underlined in italics.

| Samples | | Quality | | | Mapping | | | | Assembly | |
| --- | --- | --- | --- | --- | --- | --- | --- | --- | --- | --- |
| Platform | Name | % ≥ Q30 | Forward % ≥ Q30 | Reverse % ≥ Q30 | % reads mapped | Non-viral reads* | Avg. depth of coverage | SNP counts | N50 | Max. contig |
| MiSeq | Polio_sample_1 | 92.7 | 96.2 | 89.2 | 11.2 | 55% bacterial, 30% human | 1,235x | 3 | 7,482 | *7,482* |
|  | Polio_sample_2 | 94.6 | 95.9 | 93.2 | 14.3 | 69% bacterial, 13% human | 1,181x | 3 | 7,483 | *7,483* |
|  | Polio_sample_3 | 94.3 | 94.9 | 93.8 | 23.1 | 67% bacterial, 7% human | 1,548x | 1 | 7,483 | *7,483* |
|  | Polio_sample_4 | 95.0 | 96.4 | 93.7 | 25.4 | 69% bacterial, 4% human | 2,588x | 1 | 7,459 | *7,459* |
|  | Polio_sample_5 | 95.5 | 95.6 | 95.3 | 33.8 | 63% bacterial, 2% human | 3,058x | 1 | 7,458 | *7,458* |
|  | Polio_sample_6 | 94.6 | 96.3 | 92.9 | 38.4 | 48% bacterial, 12% human | 3,756x | 2 | 7,164 | 7,164 |
|  | Polio_sample_7 | 95.7 | 96.2 | 95.2 | 40.7 | 53% bacterial, 4% human | 3,542x | 0 | 1,308 | 1,456 |
|  | Polio_sample_8 | 93.1 | 95.8 | 90.5 | 26.1 | 67% bacterial, 5% human | 1,815x | 1 | 7,480 | *7,480* |
|  | MiSeq Average | 94.4 | 95.9 | 93.0 | 26.6 | n/a | 2,340x | n/a | 6,610 | 6,680 |
| iSeq | Polio_sample_1 | 94.0 | 94.7 | 93.2 | 11.4 | 55% bacterial, 30% human | 1,156x | 3 | 7,229 | 7,229 |
|  | Polio_sample_2 | 93.5 | 94.1 | 92.9 | 14.5 | 69% bacterial, 13% human | 1,025x | 2 | 7,485 | *7,485* |
|  | Polio_sample_3 | 93.9 | 93.9 | 93.8 | 23.8 | 66% bacterial, 7% human | 2,031x | 1 | 7,403 | *7,403* |
|  | Polio_sample_4 | 94.1 | 94.8 | 93.4 | 26.0 | 68% bacterial, 4% human | 2,164x | 1 | 7,490 | *7,490* |
|  | Polio_sample_5 | 93.7 | 94.3 | 93.1 | 33.8 | 63% bacterial, 5% human | 2,720x | 1 | 7,485 | *7,485* |
|  | Polio_sample_6 | 93.8 | 94.5 | 93.0 | 38.4 | 48% bacterial, 12% human | 2,927x | 1 | 7,478 | *7,478* |
|  | Polio_sample_7 | 93.5 | 94.3 | 92.6 | 41.3 | 53% bacterial, 4% human | 2,854x | 0 | 7,483 | *7,483* |
|  | Polio_sample_8 | 93.3 | 93.9 | 92.7 | 26.2 | 67% bacterial, 5% human | 1,428x | 1 | 7,485 | *7,485* |
|  | iSeq Average | 93.7 | 94.3 | 93.1 | 26.9 | n/a | 2,038x | n/a | 7,450 | 7,450 |

*Non-viral reads were quantified using Kraken2 and assembled to *Mycoplasma* spp. in VPipe.

**Table S13.** P-value scores of MiSeq to iSeq quality statistics for untrimmed, unscreened SARS-CoV-2, norovirus, and poliovirus datasets. Scores were calculated using Wilcoxon Rank Sum test with significance level at 0.01.

|  | Quality | | | | | Mapping | Assembly | |
| --- | --- | --- | --- | --- | --- | --- | --- | --- |
|  | % ≥ Q30, paired reads | %≥Q30, forward reads | %≥Q30, reverse reads | Read length | Insert length | % Reads | N50 | Max. Contig |
| SARS-CoV-2 | 0.0078 | 0.016 | 0.0078 | n/a | 0.013 | 0.79 | 0.0070 | 0.010 |
| norovirus | 0.13 | 0.031 | 0.016 | n/a | 0.40 | 0.016 | 0.078 | 0.13 |
| poliovirus | 0.25 | 0.022 | 0.0078 | n/a | 0.014 | 0.93 | 0.0078 | 0.0078 |

**Table S14.** P-value scores of MiSeq to iSeq quality statistics after SARS-CoV-2, norovirus, and poliovirus reads were trimmed through Trimmomatic without host screening. Scores were calculated using Wilcoxon Rank Sum test with significance level at 0.01.

|  | Quality | | | | | Mapping | Assembly | |
| --- | --- | --- | --- | --- | --- | --- | --- | --- |
|  | % ≥ Q30, paired reads | %≥Q30, forward reads | %≥Q30, reverse reads | Read length | Insert length | % Reads | N50 | Max. Contig |
| SARS-CoV-2 | 0.0078 | 0.0078 | 0.0078 | 0.0078 | 0.014 | 0.79 | 0.95 | 0.74 |
| norovirus | 0.016 | 0.016 | 0.016 | 0.016 | 0.020 | 0.62 | 0.47 | 0.30 |
| poliovirus | 0.15 | 0.0078 | 0.87 | 0.0083 | 0.013 | 0.034 | 0.15 | 0.15 |
